# Effects of far infrared therapy on arteriovenous fistulas in hemodialysis patients: a meta-analysis

**DOI:** 10.1080/0886022X.2017.1361835

**Published:** 2017-08-14

**Authors:** Qingsong Wan, Shikun Yang, Li Li, Fenfen Chu

**Affiliations:** a Department of Nephrology, The First Affiliated Hospital of the University of South China, Hengyang, Hunan Province, China;; b Department of Nephrology, The Third Xiangya Hospital of Central South University, Changsha, Hunan Province, China;; c Department of Medicine, Hunan Environment biological Polytechnic, Hengyang, Hunan Province, China

**Keywords:** Far infrared, hemodialysis, arteriovenous fistulas, meta-analysis

## Abstract

**Background:** Far infrared (FIR) therapy may have a beneficial effect on maturity and function of arteriovenous fistulas (AVFs) in hemodialysis (HD) patients. Therefore, we performed this pooled analysis to assess the protective effects of FIR therapy in HD patients.

**Methods:** The randomized controlled trials (RCTs) and quasi-RCTs of FIR therapy for HD patients were searched from multiple databases. Relevant studies were screened according to the predefined inclusion criteria. The meta-analyses were performed using RevMan 5.2 software (The Cochrane Collaboration, Oxford, UK).

**Results:** Meta-analysis showed that FIR therapy could significantly increase the vascular access blood flow level (MD, 81.69 ml/min; 95% CI, 46.17–117.21; *p* < .001), AVFs diameter level (MD, 0.36 mm; 95% CI, 0.22–0.51; *p* < .001), and the primary AVFs patency (pooled risk ratio = 1.24; 95% CI, 1.12–1.37, *p* < .001). In addition, therapy with FIR ray radiation could decrease AVFs occlusion rates (pooled risk ratio = 0.20; 95% CI, 0.08–0.46; *p* < .001) and the level of needling pain (pooled risk ratio = 0.08; 95% CI, 0.06–0.10, *p* < .001).

**Conclusions:** FIR therapy can reduce AVFs occlusion rates and needling pain level, while significantly improve the level of vascular access blood flow, AVFs diameter and the primary AVFs patency.

## Introduction

End-stage renal disease (ESRD) is a major health problem worldwide, which is a clinical syndrome resembling systemic poisoning, due to the retention of various uremic toxins. Hemodialysis (HD) is introduced in the late 1950s, it is one of effective renal replacement therapies targeted at removing uremic toxins [1]. In recent years, remarkable advances in HD treatment have been achieved, and HD is the most commonly and effectively used modality for chronic renal replacement [2]. Good and efficient vascular access is necessary for long-term HD therapy. Arteriovenous fistulas (AVFs) are an ideal choice for the uremia patients who need a permanent vascular access.

AVFs was introduced over five decades ago and have been used extensively to provide vascular access for patients requiring HD, it has lower rates of complications (e.g., infection) in comparison with other modes of HD access (i.e., temporary central venous catheters) and are the preferred method of vascular access in HD patients [3]. Due to the continual increase of ESRD patients, AVFs will continue to be a necessary and effective tool in the coming years. Unfortunately, only 60% of AVFs will be functional at 12 months [4], various therapeutic strategies for improving clinical efficiency of AVFs have been developed to overcome this shortage. Recently, a new far infrared (FIR) therapy was used for promoting the maturation of AVFs, with the double goals of increasing wound healing and increasing vascular access blood flow. In 2014, a meta-analysis by Bashar et al. [5] concluded that FIR therapy could improve both primary and secondary AVFs patency rates, but that research only included 4 trials, which caused low reliability of experimental result. So we performed this systematic review that included much more trials to assess the effect of FIR on AVFs status in HD patients.

## Materials and methods

### Data sources and search strategy

**Figure 1.**
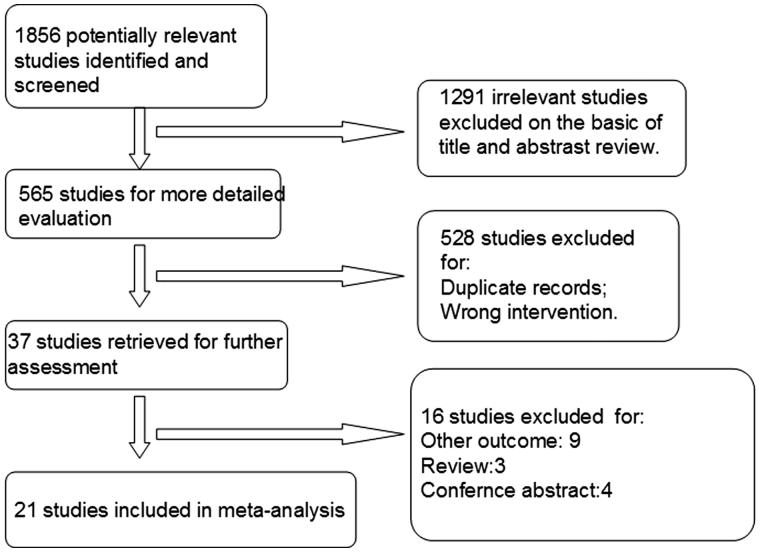
Flow diagram of study selection: far infrared therapy for hemodialysis patients.

We performed literature search on MEDLINE, EMBASE, Cochrane Central Register of Controlled Trials (CCRCT), China National Knowledge Infrastructure (CNKI), the Chinese Biomedical Literature (CBM), Wang Fang, and VIP databases (all to April 2017) to identify eligible studies. The following search terms were used: “far infra-red, far infrared, arteriovenous fistula, dialysis, hemodialysis, end-stage renal disease, renal failure, dialysis access, blood purification, kidney disease, and fistula occlusion”. The search was limited to clinical trials evaluating FIR therapy in HD patients. We present reasons for study exclusion in Figure 1.

### Inclusion criteria

Types of studies: published reports of randomized controlled trials (RCTs) or quasi-RCTs evaluating FIR therapy in HD patients with available data for our prescribed outcomes in which the language was limited to English and Chinese.

Type of participants: the studies were restricted to any patients who were diagnosed with ESRD requiring regular HD therapy using AVFs.

Type of interventions: studies comparing FIR therapy with placebo for HD patients.

Type of outcome measures: fistula maturation parameters: primary AVFs patency, access blood flow, AVFs inner diameter; fistula occlusion; puncture needle pain.

### Data extraction and assessment of study quality

Two reviewers (Q.S.W. and S.K.Y.) extracted the characteristic data of included studies independently and cooperatively. Outcomes included the level of Primary AVFs patency, access blood flow, AVFs inner diameter; fistula occlusion; puncture needle pain. Two authors (S.K.Y. and Q.S.W.) independently assessed the study quality using the Cochrane risk of bias tool [6]. The following items were assessed: (1) selection bias: Was allocation adequately concealed? Was there adequate sequence generation? (2) Detection bias: Was knowledge of the allocated interventions adequately prevented? (3) Attrition bias: Was incomplete outcome data adequately addressed? (4) Reporting bias; (5) other bias.

### Data analysis

Pooled-analysis was performed using Review Manager (version 5.2) (The Cochrane Collaboration, Oxford, UK). For continuous data using the same unit, the mean difference (MD) with 95% CI was calculated. While the pooled risk ratios were calculated for dichotomous data. The chi-squared test for heterogeneity (defined as significant when *I* ^2^ is >50%) was performed. The random effect model was used when there was significant heterogeneity; in addition, the funnel plot was analyzed to evaluate publication bias.

## Results

### Study selection

As shown in Figure 1, 1856 studies were identified after electronic searching from multiple databases, of which 1291 studies irrelevant to this study were excluded after initial assessment. Full-texts of the remaining 37 articles were retrieved for further review. At last, 21 eligibility trials were included in this meta-analysis [7–27].

### Study characteristics

The main characteristics of included trials are summarized in Table 1. The studies varied in sample size (32–280 patients). These 21 studies involved a total of 1899 HD patients, of whom 960 were treated with FIR and 939 were treated with placebo. Mean age of study participants ranged from 41.8 to 71.4 years, with a study duration of 3 weeks–12 months. Different techniques for delivering of FIR rays were used in included studies. WS TY101 FIR emitter (WS Far Infrared Medical Technology Co., Ltd., Taipei, Taiwan) was the most commonly used type which generates FIR rays with wavelengths between 5 and 25 µm. The FIR radiator was set at a height of 20 cm above the AVF with the treatment time of 40 min during HD.

**Table 1.** Characteristic of included studies.

Study	Study design	No. of patients	No. of male patients	Mean age (years)	Time of FIR administration	Frequency of HD	Time on HD (month)	Study duration (month)	Results
Choi et al. [8]	Prospective controlled trial	F:25 C:25	F:10 C:13	F:52.6 ± 10.7 C:54.0 ± 11.0	40 min during HD	3/week	F:68.3 ± 62.4 C:42.5 ± 47.7	12	FIR therapy increased the access blood flow from 881.6 to 934.7 ml/min, and improved the needling pain scores from 4 to 2
Lai et al. [14]	Prospective, randomized, controlled trial	F:118 C:98	F:50 C:36	F:62.7 ± 10.9^a^ C:63.1 ± 12.5^a^ F:67.8 ± 15.7^b^ C:66.9 ± 9.7^b^	40 min during HD	3/week	F:4.2 ± 3.5^a^^,^^c^ C:4.9 ± 4.7^a^^,^^c^ F:5.7 ± 5.6^b^^,^^c^ C:5.7 ± 5.1^b^^,^^c^	12	FIR therapy did not produce a statistical difference in unassisted patency rate at 1 year compared with the control group (25.0% versus 18.4%)
Lin et al. [17]	Prospective, randomized, controlled trial	F:72 C:73	F:37 C:38	F:61.9 ± 14.4 C:59.2 ± 15.0	40 min during HD	3/week	F:85.2 ± 41.1 C:79.2 ± 42.2	12	FIR can decrease the incidence of AVF malfunction compared with the control group (12.5% versus 30.1%), and increase the unassisted patency rate (85.9% versus 67.6%)
Lin et al. NDT [18]	Randomized, controlled trial	F:139 C:141	F:79 C:71	F:61.3 ± 14.1 C:62.8 ± 15.9	40 min during HD	3/week	F:66.0 ± 59.1 C:75.9 ± 58.0	12	FIR can improve the unassisted patency rate compared with the control group (87.4% versus 72.5%)
Lin et al. ****AJKD [19]	Randomized, controlled trial	F:60 C:62	F:32 C:35	F:63.2 ± 18.5 C:63.0 ± 14.4	40 min during HD	3/week	NR	12	FIR improves the access flow, improve the unassisted patency rate compared with the control group (87% versus 70%), decrease AVF malfunction (12% versus 29%)
Chen et al. [7]	Randomized, controlled trial	F:35 C:36	F:22 C:20	F: 62.76 ± 13.89 C: 64.21 ± 13.36	40 min during HD	3/week	NR	3 weeks	FIR could improve the AVFs access blood flow compared with the control group (245.43 ml/min versus 208.47 ml/min)
Fen et al. [9]	Prospective controlled trial	F:43 C:43	F:23 C:24	F:49.0 ± 12.0 C:47.0 ± 11.0	40 min during HD	3/week	F:2.3 ± 1.9^d^ C:2.4 ± 1.9^d^	6	FIR therapy could improve the AVFs access blood flow compared with the control group (1114 ml/min versus 921 ml/min)
Feng et al. [10]	Randomized, controlled trial	F:35 C:35	F:21 C:20	F:41.8 ± 1.50 C:43.2 ± 1.35	45 min during HD	2/week	NR	2	FIR improves the achievement ratios of once AVFs puncture compared with the control group (94.3% versus 82.9%)
He et al. [11]	Retrospective controlled trial	F:27 C:20	M:21 F: 26	52 ± 5.5	40 min during HD	3/week	NR	3 weeks	FIR improves the diameter of radial artery and cephalic vein
Ji et al. [12]****	Randomized, controlled trial	F:38 C:38	M:49 F: 27	57 ± 4	40 min during HD	3/week	NR	6	FIR decreases the rate of AVFs occlusion
Jiang [13]	Randomized, controlled trial	F:21 C:21	M:28 F:14	58 ± 3.5	40 min during HD	3/week	NR	12	FIR improves the access blood flow and decrease the rate of AVFs stenosis (14.2% versus 38.0%)
Li et al. [15]	Randomized, controlled trial	F:50 C:50	M:58 F:42	46.23 ± 15.47	40 min during HD	3/week	NR	3	FIR decreases the rate of AVFs complications (10% versus 44%)
Li et al. [16]	Randomized, controlled trial	F:40 C:40	M:52 F:28	43.6 ± 10.5	40 min during HD	3/week	NR	2	FIR therapy did not improve the access blood flow compared with the control group (928.1 ml/min versus 952.1ml/min)
Liu [20]	Randomized, controlled trial	F:26 C:26	M:35 F:17	71.4 ± 5.2	40 min during HD	3/week	NR	8	FIR decreases the rate of AVFs complications (3.8% versus 38.5%)
Shen et al. [21]	Randomized, controlled trial	F:16 C:16	M:21 F:11	30–67	30 min during HD	3/week	NR	1	FIR improves the diameter of cephalic vein (6.13 mm versus 5.67 mm) and the access blood flow (799 ml/min versus 720 ml/min)
Shen et al. [22]	Randomized, controlled trial	F:20 C:20	F:14 C:12	F:60.2 ± 15.6 C:60.1 ± 15.2	40 min during HD	3/week	F:59.5 ± 39.3 C:60.1 ± 38.9	6	FIR improves the access blood flow and decrease inflammation state
Wang et al. [24]	Randomized, controlled trial	F:66 C:66	F:30 C:33	F:52.3 ± 16.3 C:51.8 ± 14.2	40 min during HD	3/week	NR	3	FIR relives needling pain and decrease the rate of AVFs complications
Wang et al. [23]	Randomized, controlled trial	F:20 C:20	M:23 F:17	26–80	40 min during HD	3/week	NR	3	FIR decrease the rate of AVFs complications and improve the achievement ratios of once AVFs puncture compared with the control group(99.04% vs.96.92%)
Xiao and Wang [25]	Randomized, controlled trial	F:39 C:38	F:23 C:20	F:62.8 ± 16.3 C:62.2 ± 14.1	40 min during HD	3/week	NR	3	FIR improves the diameter of cephalic vein (4.7 mm versus 4.2 mm) and the access blood flow (574.2 ml/min versus 523.2 ml/min)
Yan et al. [26]	Randomized, controlled trial	F:50 C:51	F:25 C:26	F:59.2 ± 15.0 C:61.9 ± 14.4	40 min during HD	3/week	NR	12	FIR improves the access blood flow (1048.8 ml/min versus 927.5 ml/min)
Yang et al. [27]	Randomized, controlled trial	F:20 C:20	F:14 C:12	F:60.2 ± 15.6 C:60.1 ± 15.2	40 min during HD	3/week	F:5.9 ± 2.6^d^ C:5.8 ± 2.7^d^	6	FIR improves the access blood flow and decrease inflammation

F: far-infrared therapy; C: control; FIR: far infrared; HD: hemodialysis; NR: not reported.

a In AVF population.

b In AVG population.

c In years.

d Values were reported as median (range).

### Risk of bias in included studies

Allocation: on the whole, randomization was incompletely described in the included trials, some randomized methods such as computer-generated randomization random numbers were applied in nine studies for allocation [7,12,13,17–19,23,25,26]. None of the included trials clearly described the allocation concealment ways that were used.

Blinding: none of the included studies clearly reported on blinding.

Incomplete outcome data: five studies reported withdrawals [17–19,26,27].

Selective reporting: none of the included studies reported on adverse events.

**Figure 2.**
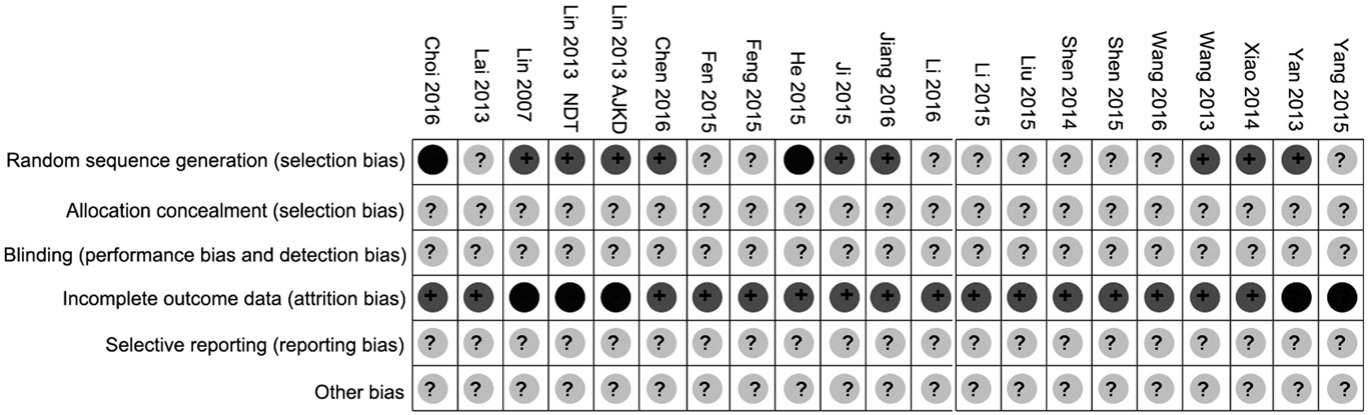
Risk of bias summary: authors’ judgments about each risk of bias item for each included study.

**Figure 3.**
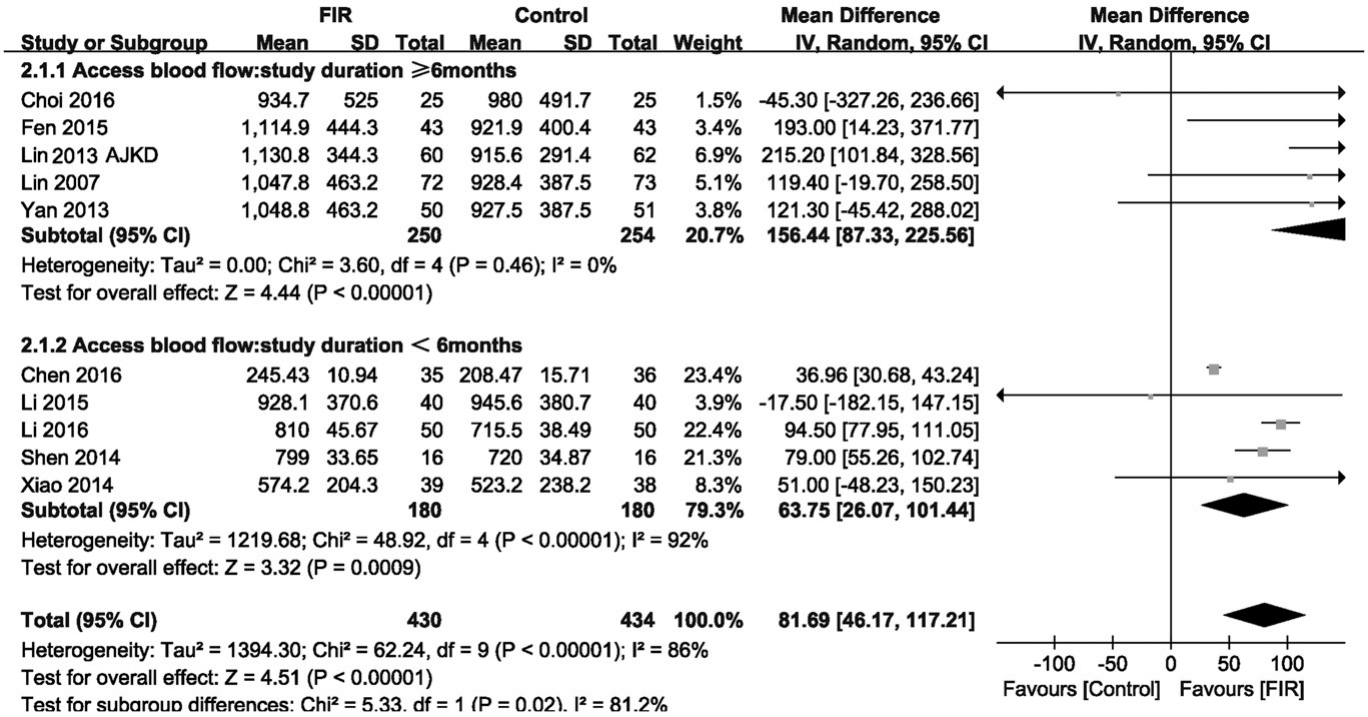
Forest plot of studies comparing the effect of far infrared therapy versus placebo on vascular access blood flow in hemodialysis patients.

Other potential sources of bias: there was insufficient information to determine if there was other potential bias in the included trials (see Figures 2 and 3).

### Effects of FIR therapy on vascular access blood flow

Ten included studies that reported changes in vascular access blood flow were analyzed under a random-effects mode (*n* = 864). The meta-analysis showed a significant increase in vascular access blood flow level in the FIR therapy group compared with that of control group (MD, 81.69 mL/min; 95% CI, 46.17–117.21; *p* < .001; Figure 3), with significant heterogeneity between studies (*p* = .00001; *I* ^2^= 86%). In addition, the subgroup analysis showed that there was no difference effect on vascular access blood flow level among trials of different duration.

### Effects of FIR therapy on AVFs diameter

**Figure 4.**
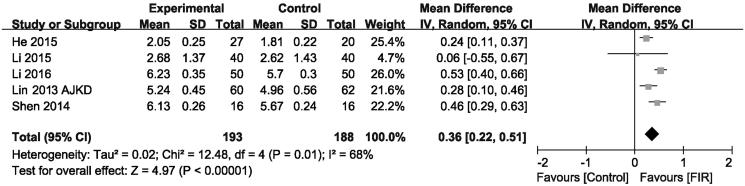
Forest plot of studies comparing the effect of far infrared therapy versus placebo on arteriovenous fistula diameter in hemodialysis patients.

As shown in Figure 4, the effect of FIR therapy on AVFs diameter was assessed in five trials (*n* = 381). Based on the results of pooled-analysis, the FIR therapy has a significant increase in AVFs diameter level compared with that of the control group (MD, 0.36 mm; 95% CI, 0.22–0.51; *p* < .001), there was evidence of heterogeneity (*p* = .01; *I* ^2^= 68%).

### Effects of FIR therapy on primary AVFs patency

**Figure 5.**
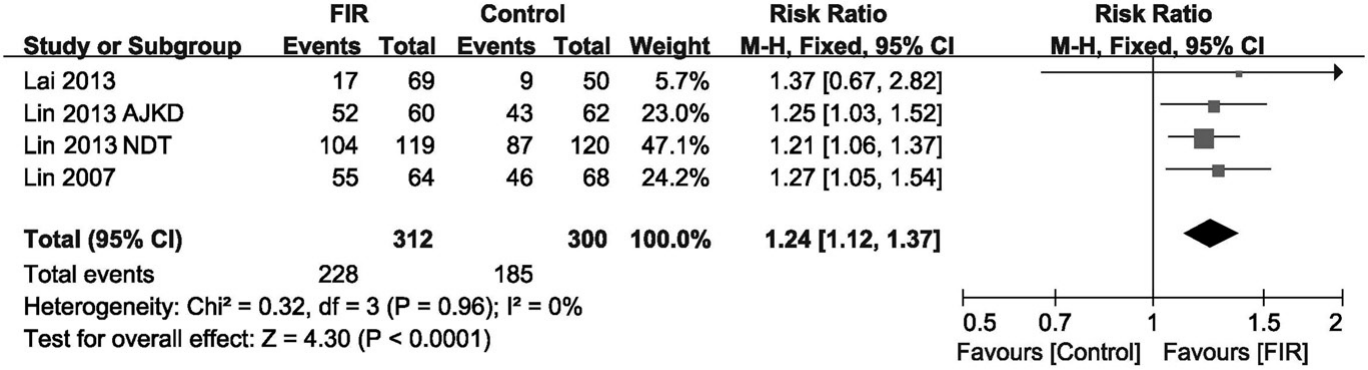
Forest plot of studies comparing the effect of far infrared therapy versus placebo on primary arteriovenous fistula patency in dialysis patients.

In line with the pooled analysis performed by Bashar et al. [5], our meta analysis assessed the effects of FIR therapy on primary AVFs patency at 12 months in four studies [14,17–19], the pooled analysis results showed significant difference between two groups, with those who received FIR therapy showing better primary patency rates compared with control (pooled risk ratio = 1.24; 95% CI, 1.12–1.37, *p* < .001), there was no evidence of statistical heterogeneity (*p* = .96; *I* ^2^ = 0%, shown in Figure 5).

### Effects of FIR therapy on AVFs occlusion

**Figure 6.**
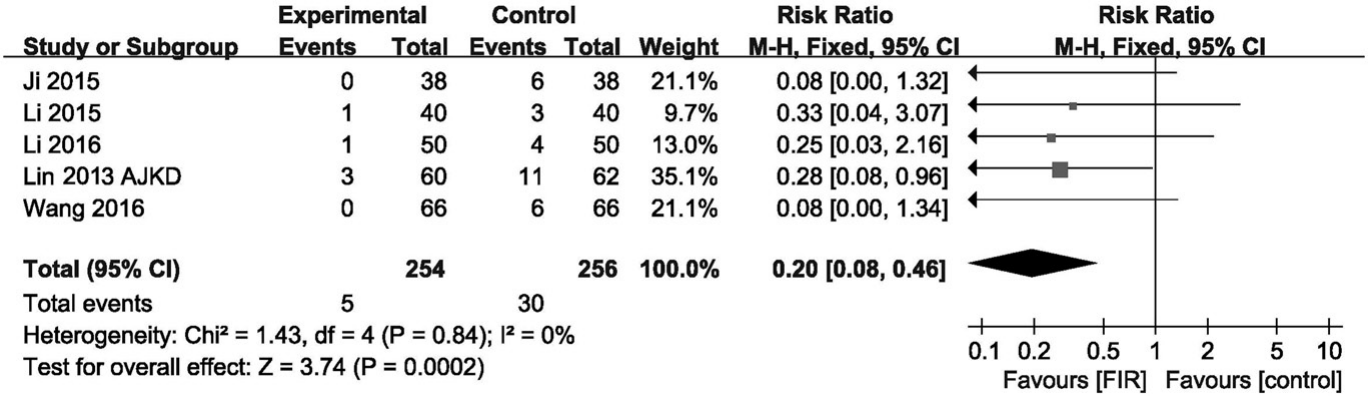
Forest plot of studies comparing the effect of far infrared therapy versus placebo on arteriovenous fistula occlusion in hemodialysis patients.

Five trials comprising 510 participants showed results for AVFs occlusion rates. Overall, therapy with FIR radiation decreased AVFs occlusion rates (pooled risk ratio = 0.20; 95% CI, 0.08–0.46) compared with that of control group (*p* < .001, Figure 6). There was no evidence of heterogeneity in these trials (*I* ^2^ =  0%, *p* for heterogeneity = .84). It is indicated that therapy with FIR ray has an advantage in reducing AVFs occlusion.

### Effects of FIR therapy on needling pain

**Figure 7.**
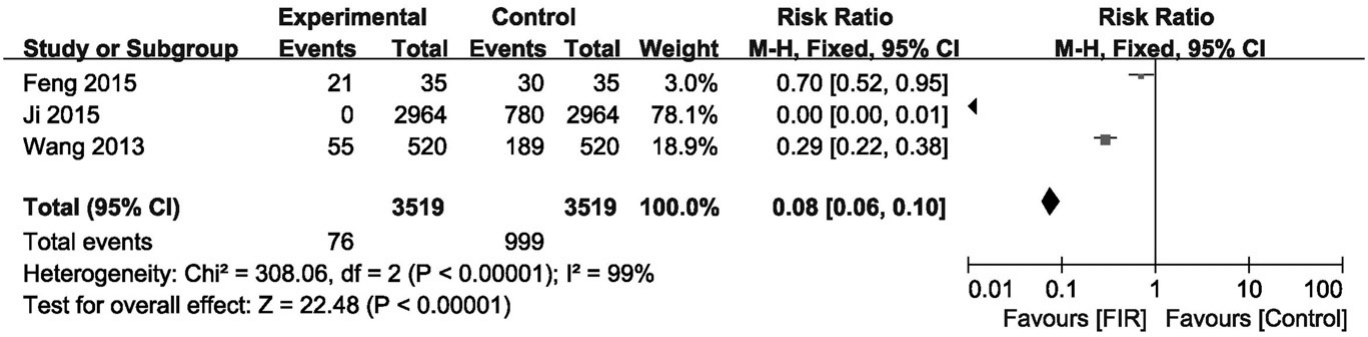
Forest plot of studies comparing the effect of far infrared therapy versus placebo on needling pain in hemodialysis patients.

Needling pain severity in patients was measured with a numeric rating scale. There are three trials reporting needling pain rates in hemodialysis. The pooled analysis result showed that the needling pain did decrease significantly in the groups receiving FIR ray radiation therapy as compared with the control group not receiving FIR ray (pooled risk ratio = 0.08; 95% CI, 0.06–0.10, *p* < .001, Figure 7). There was evidence of heterogeneity (*p* < .00001; *I* ^2^= 99%). In addition, another study not included in this pooled analysis also demonstrated that FIR therapy improved the needling pain scores from 4 to 2 after 12 months [8].

### Investigation of heterogeneity and publication bias

When we performed subgroup analysis to exploring mean change in vascular access blood flow, the analysis was stratified by study duration. In brief, there is no significant effect on vascular access blood flow (Figure 4).

Funnel plots for some key outcomes such as vascular access blood flow, AVFs diameter were asymmetric (Table 2), which suggested there was publication bias among these studies.

**Table 2.** Summary effect of far infrared therapy in trials on maintenance hemodialysis patients.

						Assessment of heterogeneity		Publication bias
Outcome variables	No. studies	No. patients	Pooled RR (95% CI)	MD change (95% CI)	*p* Value	*I*^2^ index (%)	*Q*-statistic *p* value	Funnel plots
Vascular access blood flow (ml/min) (total)	10	864	–	81.69 (46.17–117.21)	<.001	86	*p*<.00001	Asymmetric
Vascular access blood flow (ml/min) (study duration ≥6 months)	5	504	–	156.44 (87.33–225.56)	<.001	0	.46	–
Vascular access blood flow (ml/min) (study duration <6 months)	5	360	–	63.75 (26.07–101.44)	<.001	92	<.00001	–
AVFs diameter (mm)	5	381	–	0.36 (0.22–0.51)	<.001	68	.01	Asymmetric
Primary AVFs patency	4	413	1.24 (1.12–1.37)	–	<.001	0	.96	Symmetric
AVFs occlusion	5	510	0.20 (0.08–0.46)	–	<.001	0	.84	Asymmetric
Needling pain	3	–	0.08 (0.06–0.10)	–	<.001	99	<.00001	Asymmetric

RR: risk ration; MD: mean difference; AVFs: arteriovenous fistulas.

## Discussion

This pooled-analysis has consolidated data from a great deal of trials administering therapy with FIR to HD patients with AVFs. In summary, FIR therapy appears to have significantly increased AVFs diameter and improved vascular access blood flow and primary AVFs patency compared with the control group. Furthermore, FIR therapy decreased the rates of AVFs occlusion and the level of needling pain; these results suggest that FIR therapy could improve the quality of vascular access for HD.

In the beginning phase of HD development, a surgical cut down is required to access the vasculature to perform HD; this has restrained HD therapy to a great extent. In 1966, the development of a more durable AVFs by Dr. Cimino leads HD becoming more feasible [28]. Nowadays, it is estimated that there are more than 3 million patients with ESRD in Western countries [29], and two-thirds are treated with HD. Many clinical practice guidelines endorse the AVFs as the preferred form of vascular access due to it is associated with fewer complications compared to other vascular access (e.g., central venous catheter). A well-functioning AVF that constantly delivers a sufficient blood flow is a matter of critical importance for HD patients. However, the AVF has a high risk of primary failure resulting from early thrombosis and impaired maturation [4].

For vascular access to be successful, a conduit is required that has the blood flow rates of about 400 mL/min. The main causes of impaired AVFs maturation are summarized as follows: exceedingly deep location of the vein and flow diversion into accessory veins and low flow due to inflow or outflow stenosis. Numerous treatment options targeted to the cause of impaired maturation of AVFs has been developed, endovascular treatment has emerged in recent years, but these complexity intravascular interventional treatment requires doctors to be underway systemic and comprehensive treatment. Antiplatelet agents are another most popular research topic. Some trials has been demonstrated that aspirin, dipyridamole, and clopidogrel could effectively decrease AVFs thrombosis formation and improve the primary patency rates of AVFs [30].

Infrared radiation is an invisible electromagnetic wave that has a longer wave length than that of visible light, the wave length of far infrared radiation is between 5.6 and 1000 µm [31]. Some studies indicated that FIR therapy may improve endothelial function [32], in addition, animal studies have been demonstrated that FIR therapy could improve skin blood flow [33]. FIR therapy may be considered as a novel therapeutic method for improving access flow and the function of the AVFs. Our pooled analysis showed FIR therapy could significantly increase the vascular access blood flow level compared with that of the control group (*p* < .001; Figure 3). The increasing effects of FIR therapy on the vascular access blood flow may due to the thermal effect of FIR. The thermal effect of FIR results in vasodilation and increasing tissue blood flow [34]. Thermal effect induced by FIR therapy could increase AVF blood flow [34]. After 30 min of FIR therapy, skin temperature was increased to 38–39 °C; therefore, FIR therapy could avoid some side effects of thermal therapy (e.g., burn injury). There are some other possible mechanisms that may explain the non-thermal effects of FIR therapy for improving blood flow, FIR therapy could inhibit neointimal hyperplasia, decrease oxidative stress, suppress inflammation, and improve endothelial function. In addition, the beneficial effect of FIR therapy on blood flow of AVFs may be related to the activation of l-arginine/nitric oxide pathway [33].

Intimal hyperplasia (IH) is the main pathologic lesion in AVFs [35], it is characterized by an abundance of contractile smooth muscle cells, myofibroblasts, fibroblasts, and macrophages, which eventually narrow the venous outflow [36]. In addition, vascular inflammation also contributes to vascular access stenosis [37]. Thus, it can be seen that pharmacological treatments targetedly suppress IH and inflammation that are potential strategies to improve clinical outcomes of AVFs. Our pooled analysis showed that FIR therapy could significantly increase the AVFs diameter and the primary AVFs patency, while decreased the AVFs occlusion compared with that of the control group (*p* < .01; Figures 4–6). Heme oxygenase-1 (HO-1) is a known vasodilator and, at the same time, it could inhibit the proliferation of vascular smooth muscle cells, then leading to favorable conditions for maturation of AVFs. On one hand, previous research performed by Tu et al. has been shown that FIR therapy could increase the level of HO-1 expression [38]. Also, Kipshidze et al. [39] found that non-ablative infrared laser could inhibit neointimal hyperplasia. On the other hand, the anti-inflammatory effect of FIR therapy has been demonstrated, the concentration of inflammatory markers was significantly decreased in HD patients treated with FIR [40]. It indicates that the protective effects of FIR therapy for AVFs may be resulted from upregulation of HO-1 expression and anti-inflammation.

There are several important potential study limitations to this pooled analysis. First, the included studies were small scale, and some trials included in this meta-analysis were of poor quality. Second, some trials were published in Chinese, which might induce publication bias. Third, most of the included studies just reported short-term (less than 1 year) outcomes of FIR therapy, the long-term efficacy of FIR treatment need to be proven by further long-term studies, and whether some factors (e.g., gender, age, race) affects the FIR therapy curative effect has not been investigated in the included trials. Finally, there was evidence of heterogeneities in these included studies. We tried to control some of these differences by performing subgroup analysis and using random-effect models; however, we should admit that it might influence the accuracy of this pooled analysis.

## Conclusions

Taken together, using of FIR combined with conventional hemodialysis has a more beneficial effect on the function of AVFs in HD patients. However, the clinical application of FIR therapy should be concerned with more long-term and well-designed multi-center studies to evaluate the clinical value of FIR as an additional therapeutic option for HD patients. Further more laboratory-based research is required to enhance our understanding of FIR aimed at reducing IH and inhibiting inflammation to improve outcomes of AVFs.
